# Hyperketonemia Predictions Provide an On-Farm Management Tool with Epidemiological Insights

**DOI:** 10.3390/ani11051291

**Published:** 2021-04-30

**Authors:** Ryan S. Pralle, Joel D. Amdall, Robert H. Fourdraine, Garrett R. Oetzel, Heather M. White

**Affiliations:** 1 School of Agriculture, University of Wisconsin-Platteville, Platteville, WI 53818, USA; praller@uwplatt.edu; 2 VAS, Madison, WI 53718, USA; Joel.Amdall@vas.com (J.D.A.); rhfourdr@ncsu.edu (R.H.F.); 3 Department of Animal and Dairy Sciences, University of Wisconsin-Madison, Madison, WI 53706, USA; 4 School of Veterinary Medicine, University of Wisconsin, Madison, WI 53706, USA; gary.oetzel@wisc.edu

**Keywords:** ketosis, transition dairy cow, metabolic health, comorbidities, management

## Abstract

**Simple Summary:**

In dairy cows, the transition to lactation period is metabolically challenging. Elevated blood ketone bodies, known as hyperketonemia or ketosis, is a postpartum metabolic disorder that is associated with negative energy balance, greater comorbidity risk, and decreased milk production. Research to understand the etiology of hyperketonemia has highlighted risk factors and unfavorable outcomes; however, analysis of real-world data is valuable for determining the outcomes across a region. Dairy herd improvement data from herds with diverse size and production were analyzed to determine potential risk factors for and production outcomes of hyperketonemia in the Midwest region (US). Cows predicted to have hyperketonemia had greater previous lactation dry period length, somatic cell count, and dystocia, which may represent risk factors for ketosis. Cows with predicted hyperketonemia had lower milk yield and milk protein but greater milk fat and somatic cell count in the current lactation. Culling rate within 60d of calving, days open, and artificial inseminations were all greater in cows predicted to have hyperketonemia. Prevalence of hyperketonemia decreased linearly in herds with greater rolling herd average milk yield. This work demonstrates the impact of hyperketonemia on production variables which underscores the importance on continued work to reduce hyperketonemia prevalence.

**Abstract:**

Prediction of hyperketonemia (HYK), a postpartum metabolic disorder in dairy cows, through use of cow and milk data has allowed for high-throughput detection and monitoring during monthly milk sampling. The objective of this study was to determine associations between predicted HYK (pHYK) and production parameters in a dataset generated from routine milk analysis samples. Data from 240,714 lactations across 335 farms were analyzed with multiple linear regression models to determine HYK status. Data on HYK or disease treatment was not solicited. Consistent with past research, pHYK cows had greater previous lactation dry period length, somatic cell count, and dystocia. Cows identified as pHYK had lower milk yield and protein percent but greater milk fat, specifically greater mixed and preformed fatty acids (FA), and greater somatic cell count (SCC). Differential somatic cell count was greater in second and fourth parity pHYK cows. Culling (60d), days open, and number of artificial inseminations were greater in pHYK cows. Hyperketonemia prevalence decreased linearly in herds with greater rolling herd average milk yield. This research confirms previously identified risk factors and negative outcomes associated with pHYK and highlights novel associations with differential SCC, mixed FA, and preformed FA across farm sizes and production levels.

## 1. Introduction

Use of data to predict and to diagnose dairy cattle health events has become of great interest as the availability of data sources on-farm increases. Traditional laboratory and cowside diagnostics are invasive, expensive, and laborious, emphasizing the value of less invasive, higher throughput management and diagnostic tools. Use of sensors, cow- and farm-level data, and more comprehensive analysis of routinely analyzed milk samples have generated datasets that can be used to monitor and predict productivity, animal health, and inform management decisions as previously reviewed [1,2,3]. In addition to providing diagnostic and management feedback when applied, broad implementation of these data-based tools result in datasets that can provide valuable opportunities for epidemiological analysis.

Prediction of hyperketonemia (HYK), also known as subclinical or clinical ketosis, is one example target for development of data-based predictions. Postpartum HYK has a global prevalence of 15 to 22% [4,5,6] and an average cost of $289 per case due to both direct cost and indirect cost of comorbidities [7]. Given the known negative impacts of HYK on farm economics and animal health, there has been strong interest in high-throughput data-based methods of predicting herd- or cow-level HYK. Milk Fourier-transform infrared spectroscopy (FTIR) based testing for ß-hydroxybutyrate (BHB) concentration during routine milk testing is a commonly employed method for predicting ketosis; however, predicted milk BHB concentrations have lower correlations with blood BHB compared to more comprehensive models developed to predict HYK based on both milk and cow variables using approaches ranging from multiple linear regression to more advanced artificial neural networks [4,8,9,10,11,12,13].

Analysis of milk samples from routine herd testing, unless conducted more often than monthly, have limited value in diagnosing HYK in individual cows. Some cows with HYK may have developed and resolved the problem before their milk was tested and only about half of the postpartum cows will have a routine milk test fall within the primary HYK risk period. Despite these limitations, the patterns of negative associations of predicted milk BHB by FTIR [6] were similar to associations reported in controlled studies using blood BHB. Epidemiological analyses using predicted HYK via milk FTIR analysis can provide broad insight into impacts of HYK over time across regions and farms of different sizes and production levels.

One of the multiple-linear regression approaches to predicting HYK previously published [4] has been implemented within Dairy Herd Improvement (DHI) herd testing as the KetoMonitor (AgSource Cooperative, Menomonie, WI, USA) in Midwest region of the United States for several years. The use of this more robust multiple-linear regression approach to predicting HYK (using both milk sample FTIR results and cow data) represents an improved platform for conducting large-scale epidemiological studies of HYK. We hypothesize that epidemiological analysis of cow- and herd-level data generated from HYK predictions will identify HYK risk factors that are generally associated with peripartum challenges and HYK outcomes associated with shifts in nutrient partitioning reflective of negative energy balance. The objective of this study was to determine associations between predicted HYK and both risk factors and production outcomes within a dataset containing farms of varying size and production levels. To accomplish this, the current research explored the relationship between predicted HYK status and production parameters, animal health, and herd-level production. Of particular interest are several novel outcomes including milk fatty acid (FA) characteristics, differential somatic cell count, cow genetic information, farm-collected health data, and stratification of data from culled or retained cows.

## 2. Materials and Methods

Previously published [4,14] multiple linear regression predictions of HYK using test-day milk and performance variables have been implemented as a part of routine milk analysis (KetoMonitor, AgSource Cooperative, Menomonie, WI, USA) since 2014. Data from Holstein herds located within the Midwest region of the US, with at least ten months of DHI data were included in the datasets, provided that the farm granted permission for their data to be used for research, generating a dataset of convenience sampling. Data was exported in September 2020 and records from 240,714 lactations across 335 farms were included in the analysis. Data were not limited to one lactation per cow or one year per farm, so cows or farms may contribute multiple, longitudinal DHI observations.

### 2.1. Test Day Records for Milk Components and Hyperketonemia Predictions

Test day milk sample analysis and cow management records were collected from privately owned dairy farms on the day of routine herd milk sample collection as a part of the herd’s routine monthly management practices. Animal identification numbers were recorded by automatic radio-frequency identification collection or visual verification. Milk samples were collected from the morning or mid-day milkings using calibrated proportional samplers approved by the International Committee on Animal Recordings. Milk samples were collected into vials containing 2-bromo-2-nitropropane-1,3-diol (Advanced Instruments Inc., Norwood, MA, USA) for preservation and transported for analysis of milk composition according to standard test-day procedures in the laboratory of AgSource Cooperative Services (Menomonie, WI, USA). All milk samples were slowly preheated to 40 °C and mixed before analysis of milk fat and milk protein by FTIR using the Foss MilkoScan FT+ (Foss Analytical, Hillerød, Denmark) in accordance with the instrument manufacturer’s instructions and ISO 9622/IDF 141 (ISO, 2013) and AOAC International (2016) method 972.16. Analysis of somatic cell count (**SCC**) and differential SCC (**dSCC**; proportion of polymorphonuclear leukocytes and lymphocytes) was performed using Fossomatic 7 DC (Foss Analytical, Hillerød, Denmark). Milk BHB and milk acetone concentrations were predicted by FTIR using Foss Ketolab (Foss Analytical, Hillerød, Denmark). Predictions of the milk content of de novo, mixed, and preformed fatty FA in milk was based on Foss FTIR FA prediction models (Foss Analytical, Hillerød, Denmark, 2011; v1.0 and 2.0 over the course of sample analysis). Foss FTIR prediction equation version numbers used for samples tested were Foss Integrator v 2.3.8, 3.1.0, and 3.1.1 over the course of sample collection. The quality control standards and equipment calibrations were maintained by the DHI and have been described previously [4]. Briefly, per DHI standard operating procedures, milk samples were analyzed on equipment that is calibrated weekly with 12 standards, and standards are rechecked daily and hourly with a subset of 6 of the 12 standards. Intra-assay coefficients of variation for all variables were maintained at <7%. 

In line with routine procedures, on the day of sampling, cow and farm data were exported from farm herd management software, including DairyComp305 (Valley Agricultural Software, Madison, WI, USA), AgSource DM (Valley Agricultural Software, Madison, WI, USA), or BoviSync (Fond du Lac, WI, USA). Data extracted from the herd management software included previous lactation length, dry period length, gestation length, previous mature-equivalent 305-d (ME305) milk production, and age at calving. Predicted transmitting ability (PTA) were provided by the Council on Dairy Cattle Breeding (Bowie, MD, USA).

Hyperketonemia prediction models were implemented based on stratification of cows as primi- or multiparous and day in milk (DIM) of sample collection (5 to 11 DIM or 12 to 20 DIM) which was demonstrated to yield more accurate results [4]. Models were implemented by AgSource and yielded a predicted blood BHB which was used to derive a predicted hyperketonemia health (pHealth) status of predicted HYK (pHYK) or not predicted HYK (pNonHYK) using a 1.2 mM predicted blood BHB cutoff. Results were returned to farm owners as a part of the KetoMonitor (AgSource, Menomonie, WI, USA) report. No information confirming blood BHB concentration, HYK diagnosis, or subsequent treatment for HYK was solicited. 

### 2.2. Cow- and Herd-Level Data Aggregation

All data described above, as well as subsequent outcomes for each cow, were maintained by AgSource. Retrieved data were organized into two data sets for our analysis: a monthly herd-level record data set and a lactation record data set.

The herd-level data set was composed of 17,387 observations of rolling herd average (RHA) production and 12 mo pHYK prevalence data. These herd-level records are collected or updated every month and each herd contributed a range of data from 1 to 72 monthly observations. Herd-level records were excluded when the interval on DHI test was less than 365 days (*n* = 251 herd records), the number of cows eligible for health status prediction did not exceed 10 cows in the past 12 mo period (*n* = 5489 herd records), or the herd-level record had pHYK prevalence rates greater than 100% (*n* = 5 herd records). Therefore, 11,640 herd records were eligible for statistical analysis. These herd-level records were classified into quartile groups based on RHA milk yield (quartile [milk range]): quartile 1 [<11,137 kg], quartile 2 [11,137 to 12,265 kg], quartile 3 [12,266 to 13,264 kg], and quartile 4 [≥13,265 kg]. Classifying herd-level records based on these quartiles allowed for a herd to be represented in one or more quartiles over time.

The cow-level data set is composed of cow lactation records and each cow could contribute multiple records. From the 258,610 cow lactation records retrieved, non-Holstein lactation records (*n* = 10,602), duplicate records (*n* = 7142), and lactation records from herds represented by less than 11 records (*n* = 152) were removed, leaving 240,714 records for cow-level analysis. Lactation record parity number was classified as 1, 2, 3, 4, and 5+ (parity number ≥ 5). In addition to data already described above, data exported for this analysis included days open; calving interval; previous lactation SCC, somatic cell score (**SCS**), and dSCC; current lactation first SCC, SCS, and dSCC; artificial inseminations; calf mortality; twinning; calving ease; and culling reason. Calving ease was a numerical score from 1 to 5 (1: no assistance; 2: some assistance; 3: mechanical assistance; 4: cesarean section; 5: abnormal delivery). Culling reasons were injury/disease (includes physical injury, lameness, and illnesses such as DA, ketosis, metritis, retained placenta, milk fever, etc.), sold as dairy (animals sold that enter another dairy herd), death, production, or mastitis. Treatments for diseases are not recorded in a consistent, exportable manner in dairy management software and therefore could not be extracted. Lactation records were classified as being predicted HYK based on milk acetone and BHB thresholds proposed by de Roos [13]. Disease events were retrieved from farm herd management software for farms with importable records for each disease. Within those farms, lactation records without a recorded disease case before 60 DIM were assumed as non-cases. For each disease, lactation records were excluded from analysis if the respective herd had no recorded event for the disease of interest, resulting in a different number of herds and lactations in analysis of each disease. Disease events included ketosis (*n* = 10,152 of 129,957 lactations across 335 herds), displaced abomasum (DA; *n* = 3114 of 164,561 lactations across 88 herds), milk fever (*n* = 675 of 98,017 lactations across 53 herds), retained placenta (*n* = 5667 of 153,822 lactations across 79 herds), and mastitis (*n* = 7888 of 168,764 lactations across 86 herds). Cow lactation records were labeled culled if the cow was removed from the herd or died within the first 60 DIM; all other records were labeled retained. Records were considered a complete lactation (*n* = 191,722) when a cow was culled (*n* = 64,552) or dried off (*n* = 127,170; cessation of lactation).

### 2.3. Statistical Analysis

Data analysis was performed using the SAS software (version 9.4; copyright 2002 to 2012, SAS Institute Inc., Cary, NC, USA) using the GENMOD and PHREG procedures. Generalized estimating equations (GEE; GENMOD procedure) were used to analyze all responses except for time to event. Model distribution and link functions varied. For herd-level data, weighted regression analysis was performed (GENMOD procedure, SAS 9.4) to associate the pHYK prevalence (12 mo period) with RHA production variables. With pHYK prevalence as the dependent variable, GEE models were fitted (negative binomial distribution and log normal link) with an exchangeable correlation matrix with the repeated subjects of herd and year within herd as the cluster and subcluster effects, respectively. Observations were weighted based on the reciprocal number of cows with HYK prediction (12 mo period). Additionally, a GEE model was used to evaluate pHYK prevalence across RHA quartiles (fixed effect) based on the same distribution, link, and correlation matrix described for the weighted regression. A linear contrast of RHA quartile effect on pHYK was also conducted. Model effects were considered significant when *p* ≤ 0.05. Pairwise least-squares mean comparisons of RHA quartile pHYK prevalence was adjusted by Bonferroni’s method.

For cow-level data, production (e.g., milk yield, milk component yield, and previous mature equivalent yields) and predicted transmitting ability variables were evaluated with a Gaussian distribution and identity link. Milk composition variables expressed as a percentage (e.g., milk fat and protein) were evaluated with a negative binomial distribution and log link. Count data (e.g., milk SCC and SCS, calving interval, calving ease, and AI services) variables were analyzed with a Poisson distribution and log link. Incidence (or event) data (e.g., retention status, DA incidence, calf mortality) variables were analyzed with a binomial distribution and logit link. The reproductive responses AI services and days open were only evaluated on records from completed lactations exclusively. All GEE models included fixed effects of pHealth, parity, and the pHealth × parity interaction, as well as an exchangeable correlation matrix with the repeated subjects of herd and cow within herd as the cluster and subcluster effects, respectively. Additionally, peak milk yield and mature equivalent production (current lactation) responses included fixed effects of retention status and all possible higher order interactions. Exclusion of the pHealth × parity interaction effect allowed for DA incidence model convergence. The milk fever incidence model achieved convergence when pHealth was the sole fixed effect. Fragility models (FM; PHREG procedure, log normal distribution) were used to analyze time to event data, such as DIM until a disease event (e.g., DA) or DIM until peak milk yield with a hazard ratio less than 1 indicating that the event occurred later in pHYK cows. Fixed effects included pHealth, parity, and the pHealth × parity interaction with the DIM culled as a censoring variable (except for the DIM until culled response) and the random effect of herd. To achieve FM model convergence for the DIM until milk fever event, the pHealth × parity interaction was excluded.

In text, data describing fixed effects are represented as least-squares means ± SEM. Significant differences were declared when *p* ≤ 0.05. Pairwise least-squares mean comparisons were made when a fixed effect was observed (*p* ≤ 0.05). For an interaction including pHealth (e.g., pHealth × parity or pHealth × retention; *p* ≤ 0.05), simple (or “slice”) effect comparisons were made between pHYK and pNonHYK within levels of the interacting variable. Pairwise and simple effect comparison *p*-values were adjusted by Bonferroni’s method and considered significant or marginal post-adjustment using the *p*-value thresholds reported above.

## 3. Results

### 3.1. General Descriptive Statistics of the Dataset

**Figure 1:**
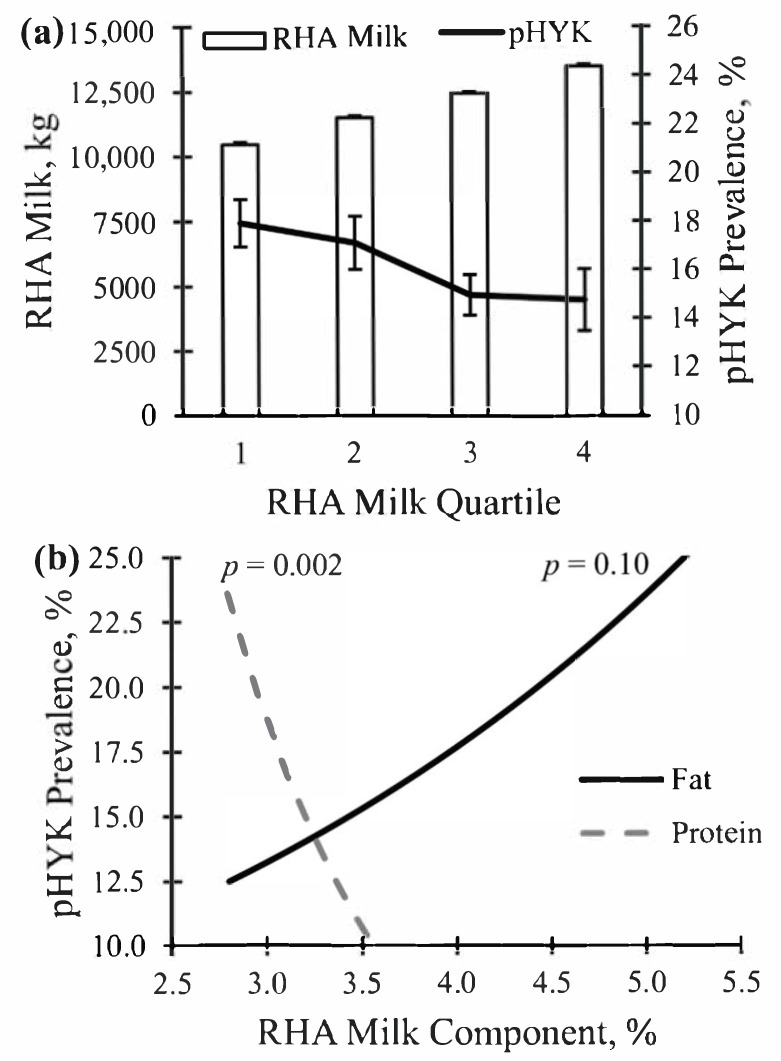
Prevalence of predicted hyperketonemia (HYK) status (**a**) across rolling herd average (RHA) milk production quartiles and (**b**) as associated with RHA milk fat and protein percent in a test day dataset of herds from the Midwest region of the United States. Prevalence of HYK was based on proportion of samples collected between 5 and 20 days in milk predicted as HYK (pHYK; predicted BHB ≥ 1.2 mM) or not (pNonHYK; predicted BHB < 1.2 mM). Herds and records per quartile: Q1: 158 and 2885; Q2: 147 and 2884; Q3: 142 and 2885; Q4: 100 and 2885. Panel (**a**): modeled linear effect of pHYK prevalence using generalized estimating equation weighted based on the number of cows (*p* = 0.02). Panel (**b**): HYK predicted prevalence was negatively associated with RHA protein % (*p* = 0.002) but there was no evidence of association for RHA fat % (*p* = 0.10).

A total of 240,714 records from test day samples collected between 5 to 20 DIM across a four-year period were analyzed. Descriptive statistics are shown in Table 1. Of all records, 174,690 were from unique cows from 335 herds. On average, 521 fresh cows were sampled per herd over the four-year period. An average of 37% of records per herd were from primiparous animals while 63% were from multiparous cows. Overall, pHYK prevalence was 15.8% and by lactation was 1, 4.0%; 2, 13.9%; 3, 26.4%; 4, 32.0%; 5+, 35.0%. The dataset included a wide range of herd sizes with a minimum of 11 and a maximum of 9550 cows per herd, with a median of 179. Overall and RHA quartile herd production demographics are shown in Table 2. Milk production RHA across contributing herds ranged from 5539.27 to 18,196.30 kg. Across RHA quartiles, linear modeled pHYK decreased linearly (*p* = 0.02) as RHA milk yield quartiles increased (Figure 1). Prevalence of predicted HYK was positively associated with RHA milk production (*p* = 0.01; data not shown) and RHA protein percent and yield (*p* < 0.001; Figure 1) but not RHA milk fat percent or yield (*p* > 0.05; Figure 1). 

### 3.2. Relationships between Prediction of Hyperketonemia and Prior Cow and Parturition Factors

**Figure 2:**
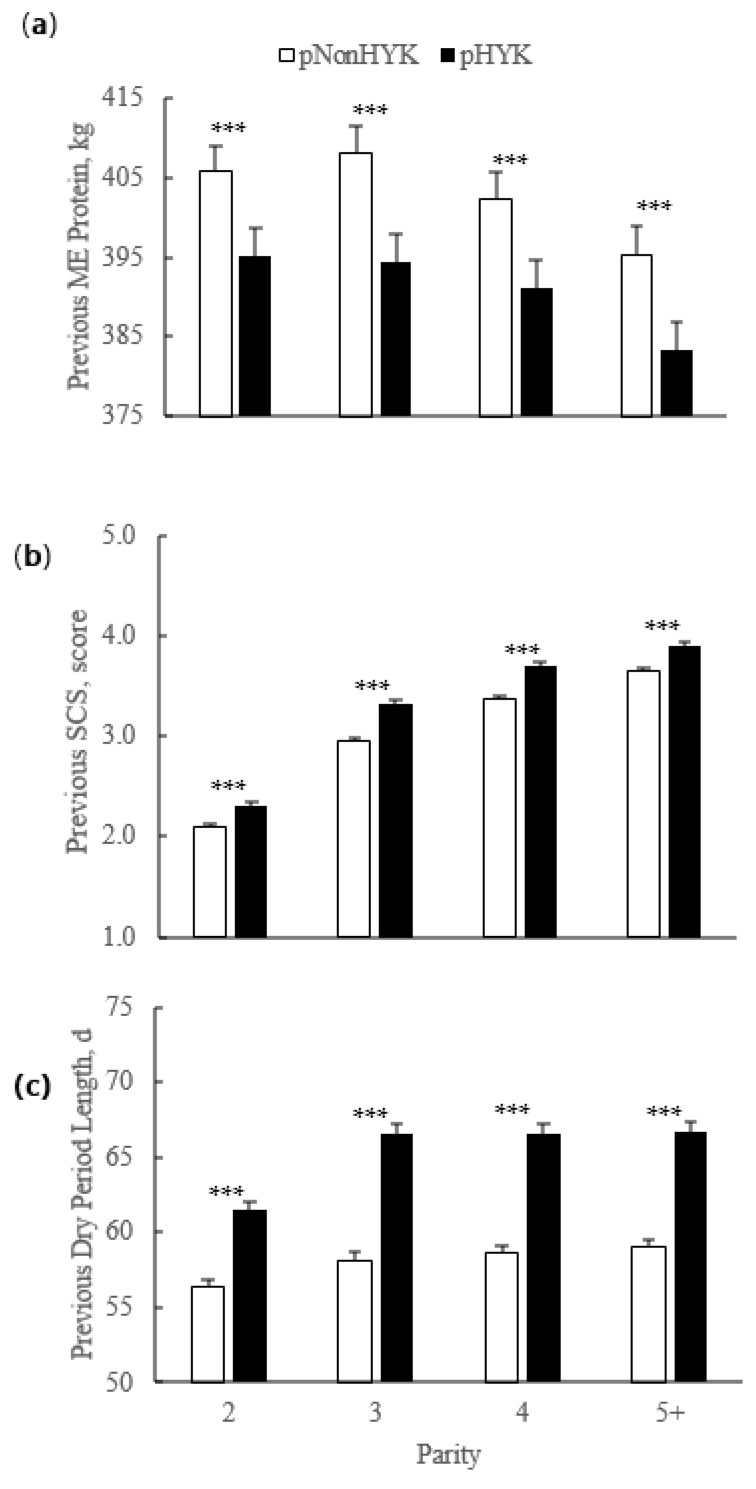
Parity by predicted hyperketonemia (HYK) status interaction on previous lactation (**a**) mature equivalent (ME) milk protein yield (kg); (**b**) somatic cell score (SCS; score); and (**c**) dry period length (d). Blood ß-hydroxybutyrate (BHB) was predicted based on test day milk sample and production variables collected between 5 to 20 days in milk. Predictions were classified as predicted HYK (pHYK; predicted BHB ≥ 1.2 mM) or not (pNonHYK; predicted BHB < 1.2 mM) for analysis. Records per lactation: 1, 88,782; 2, 67,327; 3, 43,790; 4, 23,999; 5+, 16,816. Parity by predicted HYK interactions *p* < 0.001. Asterisks (***) indicate simple effect of classification (*p* < 0.001) within parity.

For primiparous cows, calving age was older (*p* < 0.001) for pHYK (25.2 ± 0.1 months) than for pNonHYK (24.8 ± 0.1 months). For multiparous cows, previous DIM and gestation length were greater (*p* < 0.001) for pHYK than pNonHYK cows (352.2 ± 1.4 vs. 342.6 ± 1.2 d and 278.5 ± 0.2 vs. 277.8 ± 0.2 d, respectively). Previous lactation SCC was greater (*p* < 0.001) for pHYK (248.7 ± 7.0 vs. 221.9 ± 5.6). Previous lactation mature equivalent (ME) milk production (12,959.6 ± 109.5 vs. 13,294.8 ± 108.5 kg) and ME fat yield (494.4 ± 4.4 vs. 503.6 ± 4.3 kg) were less (*p* < 0.001) for pHYK cows. Somatic cell count was greater (*p* < 0.001) for pHYK cows (248.7 ± 7.0 vs. 221.9 ± 5.6 cpm × 1000). There was an interaction of parity and HYK prediction (*p* < 0.001) for previous lactation dry period length, SCS, and ME milk protein yield (Figure 2). 

**Figure 3:**
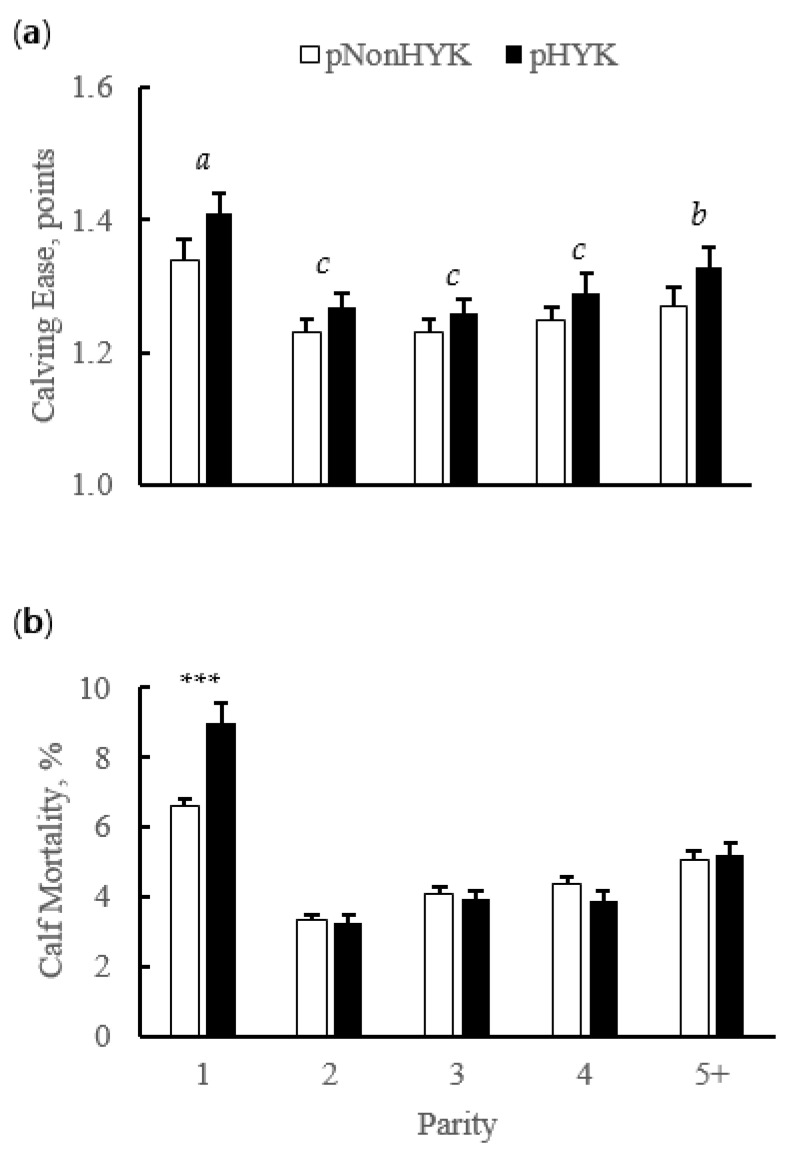
Effect of parity, predicted hyperketonemia (HYK) status, and the interaction on (**a**) calving ease (points); and (**b**) calf mortality (%). Blood ß-hydroxybutyrate (BHB) was predicted based on test day milk sample and production variables collected between 5 to 20 days in milk. Predictions were classified as predicted HYK (pHYK; predicted BHB ≥ 1.2 mM) or not (pNonHYK; predicted BHB < 1.2 mM) for analysis. Records per lactation: 1, 88,782; 2, 67,327; 3, 43,790; 4, 23,999; 5+, 16,816. Panel (**a**): predicted HYK, *p* < 0.001; parity, *p* < 0.001, differing superscript letters (*a*, *b*, *c*) indicate differences between parities; parity by predicted HYK interaction *p* = 0.48. Panel (**b**): predicted HYK, *p* = 0.25; parity, *p* < 0.001; parity by predicted HYK interaction, *p* = 0.002, asterisks (***) indicate simple effect of classification (*p* < 0.001) within parity.

Birth rate of twins did not differ (*p* = 0.34) by predicted HYK status (4.1% ± 0.2 vs. 3.9% ± 0.1 pHYK vs. pNonHYK). Calving interval was greater (*p* < 0.001) for pHYK than pNonHYK (418 ± 1 vs. 401 ± 1 days). Calving ease score was greater (*p* < 0.001) for pHYK cows and was affected by parity (*p* < 0.001). The interaction of predicted HYK status and parity affected (*p* = 0.002) calf mortality (Figure 3) and was greater for pHYK, compared to pNonHYK, first lactation cows.

### 3.3. Relationships between Prediction of Hyperketonemia and Early Lactation Performance

**Figure 4:**
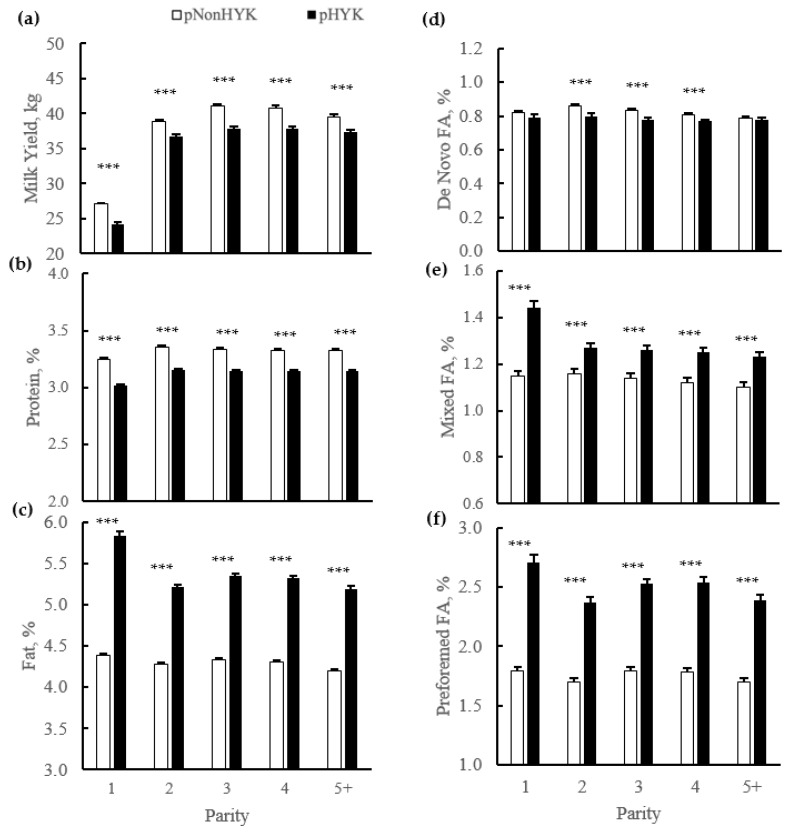
Parity by predicted hyperketonemia (HYK) status interaction on first milk test (**a**) milk yield (kg); (**b**) protein (%); (**c**) fat (%); (**d**) de novo fatty acids (FA; %); (**e**) mixed FA (%); and (**f**) preformed FA (%). Blood ß-hydroxybutyrate (BHB) was predicted based on test day milk sample and production variables collected between 5 to 20 days in milk. Predictions were classified as predicted HYK (pHYK; predicted BHB > 1.2 mM) or not (pNonHYK; predicted BHB < 1.2 mM) for analysis. Records per lactation: 1, 88,782; 2, 67,327; 3, 43,790; 4, 23,999; 5+, 16,816. Predicted HYK, *p* < 0.001; parity, *p* < 0.001; parity by predicted HYK interaction *p* < 0.001 for all panels. Symbols indicate simple effect of classification (***, *p* < 0.001) within parity.

**Figure 5:**
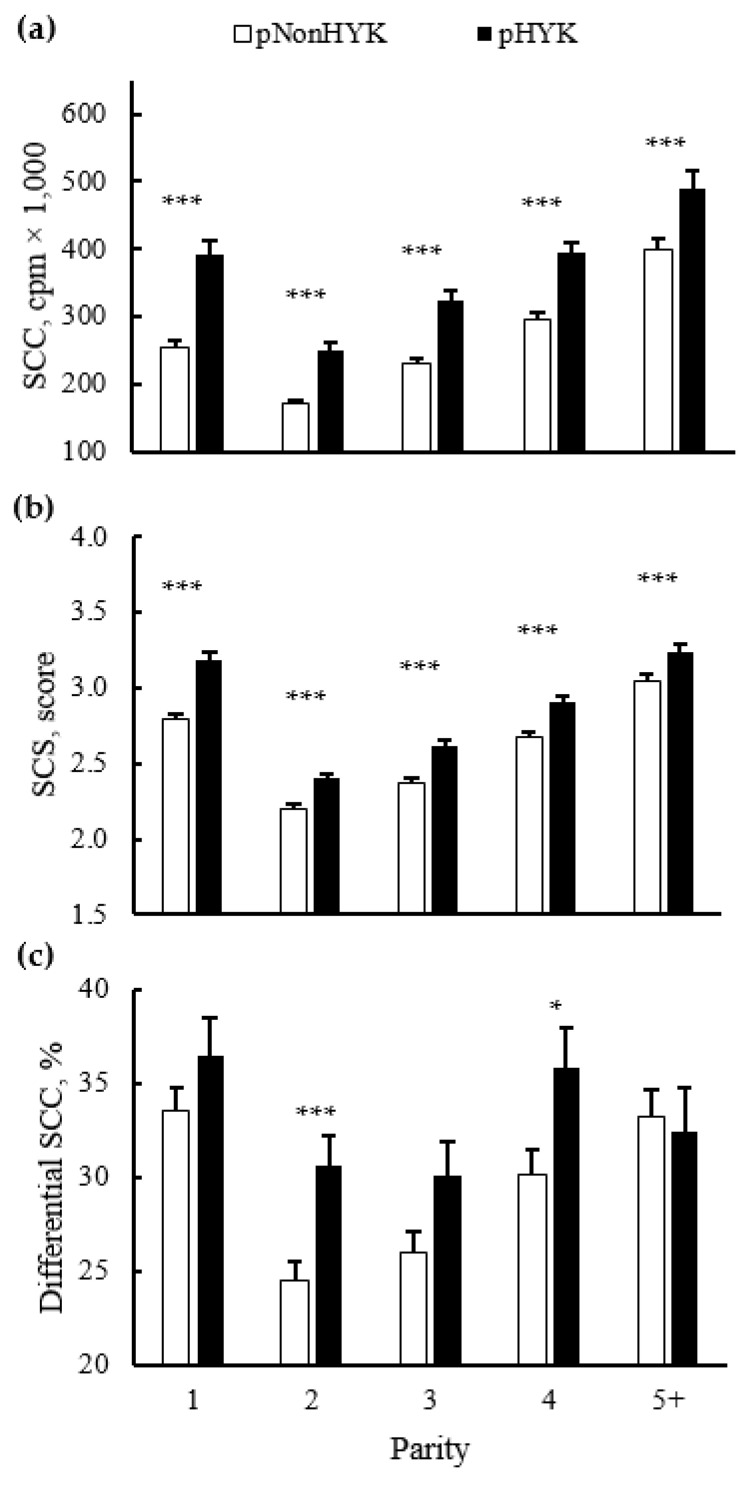
Parity by predicted hyperketonemia (HYK) status interaction on first milk test (**a**) somatic cell count (SCC; cpm × 1000); (**b**) somatic cell score (SCS; score); and (**c**) differential SCC (%). Blood ß-hydroxybutyrate (BHB) was predicted based on test day milk sample and production variables collected between 5 to 20 days in milk. Predictions were classified as predicted HYK (pHYK; predicted BHB ≥ 1.2 mM) or not (pNonHYK; predicted BHB < 1.2 mM) for analysis. Records per lactation: 1, 88,782; 2, 67,327; 3, 43,790; 4, 23,999; 5+, 16,816. Panel (**a**): predicted HYK, *p* < 0.001; parity, *p* < 0.001; parity by predicted HYK interaction *p* = 0.008. Panel (**b**): predicted HYK, *p* < 0.001; parity, *p* < 0.001; parity by predicted HYK interaction *p* = 0.001. Panel (**c**): predicted HYK, *p* < 0.001; parity, *p* < 0.001; parity by predicted HYK interaction *p* = 0.02. Symbols indicate simple effect of classification (***, *p* < 0.001; *, 0.01 < *p* < 0.05) within parity.

There were interactions of parity and predicted HYK status on all milk production and composition variables from first milk test postpartum. Although the magnitude of difference between pHYK and pNonHYK differed within lactations generating an interaction, across all parity classes the directionality of difference was similar. There was an interaction (*p* = 0.001) of pHealth and parity on milk FTIR-predicted BHB and acetone, although both were greater across lactations for pHYK cows. Milk FTIR BHB for pNonHYK vs. pHYK, respectively (parity 1, 2, 3, 4, 5+, ±SEM), were 0.060, 0.057, 0.062, 0.061, 0.055 ± 0.001 mM vs. 0.237, 0.158, 0.164, 0.161, 0.148 ± 0.003 mM. Milk FTIR acetone for pNonHYK vs. pHYK, respectively (parity 1, 2, 3, 4, 5+, ±SEM), were 0.100, 0.069, 0.078, 0.078, 0.072 ± 0.001 mM vs. 0.636, 0.260, 0.282, 0.275, 0.254 ± 0.009 mM. Percent agreement on diagnosis as pHYK and pNonHYK between linear regression models used here and FTIR milk Acetone cutoffs were 87.61% ± 0.4 and 74.88% ± 0.7 for pNonHYK and pHYK, respectively. Agreement between linear regression and FTIR milk BHB was 81.04% ± 0.7 and 74.84% ± 1.0 for pNonHYK and pHYK, respectively. First test milk yield and percent protein were less (*p* < 0.001) for pHYK while percent fat was greater (*p* < 0.001) for pHYK, compared to pNonHYK cows (Figure 4). De novo milk FA were less (*p* < 0.001) for second, third, and fourth lactation pHYK cows. Mixed and preformed FA were greater (*p* < 0.001) for pHYK cows. First test milk energy yield was greater (*p* = 0.001) for pHYK cows (29.7 vs. 29.0 ± 0.25 Mcal). Somatic cell count and SCS were greater (*p* < 0.001) for pHYK cows. Differential SCC was greater (*p* ≤ 0.02) within second and fourth lactation pHYK cows (Figure 5).

### 3.4. Relationships between Prediction of Hyperketonemia and Cow Outcomes

Prevalence of farm-recorded cases of ketosis in management software was 7.7%. Of pHYK and pNonHYK cows, 17.4% ± 0.3 and 5.9% ± 0.0 were identified as farm-recorded ketosis cases, respectively. Displaced abomasum incidence was greater (*p* ≤ 0.001) for pHYK cows (5.41% ± 0.43 vs. 1.06% ± 0.11). Within cases of DA, there was a parity by predicted HYK interaction (*p* < 0.001) for DIM until DA event to be later in pHYK cows (mean and 95% confidence interval for parity 1, 2, 3, 4, and 5+, respectively: 0.3 [0.2, 0.4], 0.5 [0.5, 0.6], 0.6 [0.5, 0.7], 0.5 [0.4, 0.6], and 0.5 [0.4, 0.6]). Milk fever prevalence was greater (*p* = 0.02) in pHYK cows (0.6% ± 0.10 vs. 0.5% ± 0.02). Retained placenta was greater (*p* ≤ 0.001) for pHYK (5.6% ± 0.7 vs. 3.7% ± 0.5). There was no impact (*p* ≥ 0.52) of pHealth on time to health event for mastitis, milk fever, or retained placenta.

**Figure 6:**
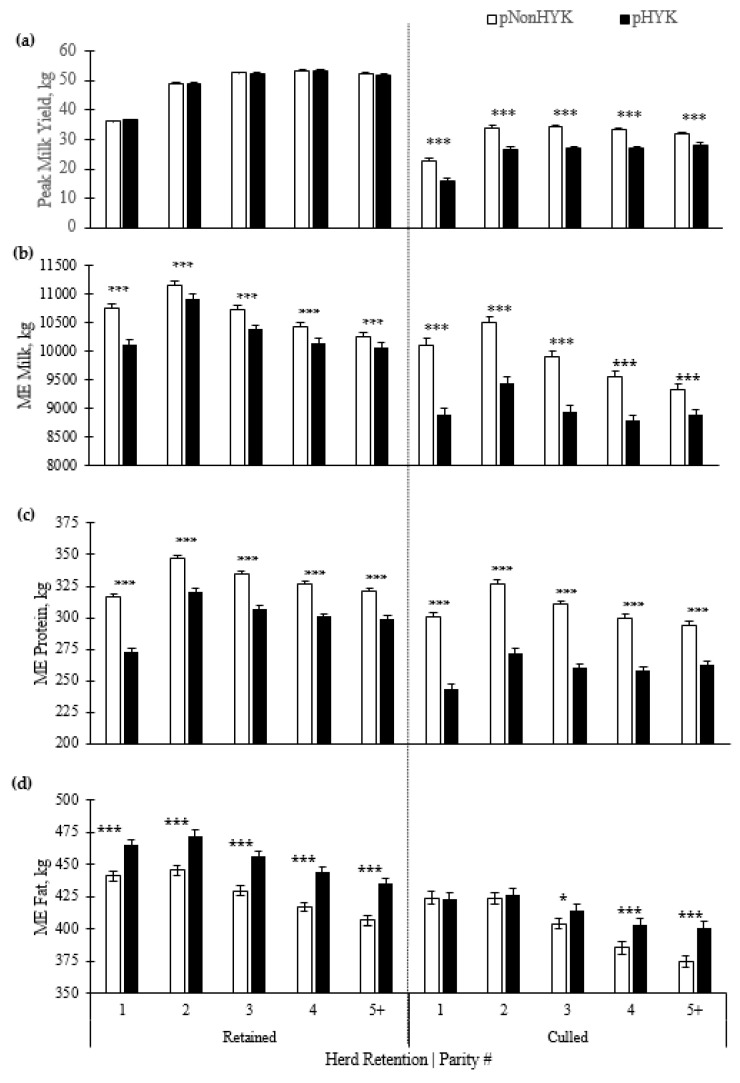
Parity by predicted hyperketonemia (HYK) status interaction within retained or culled (first 60 d) cows on (**a**) peak milk yield (kg); (**b**) mature equivalent (ME) milk (kg); (**c**) ME protein (kg); and (**d**) ME fat (kg). Blood ß-hydroxybutyrate (BHB) was predicted based on test day milk sample and production variables collected between 5 to 20 days in milk. Predictions were classified as predicted HYK (pHYK; predicted BHB ≥ 1.2 mM) or not (pNonHYK; predicted BHB < 1.2 mM) for analysis of records. Records per lactation: 1, 88,782; 2, 67,327; 3, 43,790; 4, 23,999; 5+, 16,816. Parity × predicted HYK × retention interactions *p* ≤ 0.002. Symbols indicate simple effect of classification (***, *p* < 0.001; *, 0.01 < *p* ≤ 0.05) within parity.

Parity and predicted HYK interacted (*p* = 0.003) on cows culled within 60 d postpartum, with pHYK cows having a greater percentage of cows culled across parities (Table 3). Hazard ratio for day of culling (0.7 [0.6, 0.8]) indicates that pHYK cows were culled later (parity × pHealth *p* = 0.003) across parities. Peak milk yield did not differ between pHYK and pNonHYK retained cows, although peak milk yield was less for pHYK cows that were culled within 60d (Figure 6). Retained pHYK cows peaked in milk earlier (*p* < 0.001) with an average hazard ratio of 1.1 [1.1, 1.1] across parities. Mature equivalent milk, protein, and fat were examined within the retained and culled subsets of the dataset, with all three demonstrating an interaction (*p* ≤ 0.002) of parity, predicted HYK, and retention (Figure 6).

The primary reasons for culling (90% of culls) were injury/disease, sold as dairy, death, production, and mastitis. As a percent of cows culled, death was greater (*p* < 0.001) for pHYK than pNonHYK (18.7% ± 0.9 vs. 14.7% ± 0.7). There was an interaction (*p* ≤ 0.02) between parity and predicted HYK for injury/disease, sold as dairy, and mastitis as culling reasons; however, the directionality was the same across parities. Injury/disease was listed more for pHYK cows (42.6 ± 1.6 vs. 33.4% ± 1.2). Sold as dairy and mastitis were listed less for pHYK cows than pNonHYK cows (sold as dairy: 8.5% ± 1.2 vs. 12.9% ± 1.5; mastitis: 10.6% ± 0.7 vs. 16.0% ± 0.9).

There was an interaction (*p* < 0.001) between parity and predicted HYK on days open for multiparous cows; however, days open was consistently greater (*p* < 0.001) for pHYK within each lactation and the interaction was due to the scale of difference between pHYK and pNonHYK across lactations (Table 3). Similarly, the number of artificial inseminations were influenced by the interaction (*p* < 0.001) between parity, and predicted HYK was greater (*p* < 0.001) for pHYK cows within each lactation.

Genetic PTA for milk, daughter pregnancy rate, productive life, SCS, net merit, and ketosis were affected by an interaction of parity and predicted HYK (*p* < 0.02; Table 3). There was no evidence of difference between pHYK and pNonHYK for PTA milk or PTA productive life of first and second lactation cows but both were less (*p* ≤ 0.05) in third, fourth, and fifth and greater pHYK cows. Conversely, daughter pregnancy rate was less (*p* ≤ 0.05) in pHYK cows in first, second, third, and fourth lactation cows but not different in fifth and greater lactation cows. Across all lactations, PTA SCS (*p* < 0.001) was greater and PTA net merit (*p* ≤ 0.05) was less for pHYK cows. The PTA ketosis was more negative (*p* < 0.001) for pHYK cows across all lactations.

## 4. Discussion

Application of data-based predictions of health incidences has been of increasing interest for applied dairy management practice. Although not as precise as blood diagnostics, these predictions may provide valuable tools to farms given the lower expense and labor required to execute. Of the myriad of data streams or technologies that can be monitored and used for predictions, daily milk yield and composition remains one of the most widely accepted and used by farms [15]. Unlike other options, a more comprehensive analysis of milk data does not require a capital equipment purchase at the farm-level; rather, it relies on more advanced analysis equipment at the DHI laboratory and adds value to routine DHI milk sampling. Historically, DHI milk sampling has been conducted at monthly intervals which lends itself to monitoring herd-level production and health metrics over time and across regions, especially given that management data is also collected as a part of these routine tests. Retrospectively analyzing datasets of this nature with an epidemiological approach can provide great value in determining real-world associations; however, one must remember that these relationships are associative and not causal and the absence of information regarding cow treatments or diets is a limitation. There is also a potential for bias in the epidemiological associations observed given that some of the response variables (e.g., milk yield, days dry, etc.) were included in the predictive model used for pHealth determination. Even given these limitations, models must be providing some value as a proxy measure of HYK given that differences within variable by pHealth status can be identified for comorbidities as discussed further below. For health events such as HYK, in depth understanding of etiology and causality can be examined by intense research studies. It must be noted that those experiments have limitations of their own given that HYK is sometimes induced rather than naturally occurring; research housing systems may be more controlled and decrease animal competition (e.g., tie-stalls); and inference may be limited. Taken together, determining associations in large, epidemiological datasets can inform both future research and management practices and therefore interrogation of these datasets is of value.

The analysis presented here is the first large scale analysis of data from the previously published multiple linear regression models that utilize both cow data and milk analysis to predict blood BHB with greater than 83% accuracy in Holstein cows [4]. More comprehensive prediction models, such as those used in this analysis, have been demonstrated to be more accurate than using milk FTIR-predicted BHB or acetone alone [4,12]. Cutoffs have been suggested for milk FTIR-predicted BHB and acetone [13] and they have been incorporated into more comprehensive models or considered together with other variables [4,13]. Within this dataset, using milk FTIR-predicted BHB or acetone alone had at least 80% diagnostic agreement for pNonHYK cows, but only 75% agreement for pHYK cows. Differences in diagnostic agreement results in a predicted prevalence of 25% or 34% using only milk FTIR-based acetone or BHB, respectively. It is important to note that without the gold-standard blood BHB reference, relative diagnostic accuracy cannot be determined. Milk FTIR-predicted BHB and acetone, along with other milk components, are important contributors to comprehensive pHealth models, and it is likely that continued advancements in milk FTIR analysis will continue to provide proxy measures of cow health and productivity.

The hypothesis driving this research was that epidemiological analysis of cow- and herd-level data generated from HYK predictions will identify HYK risk factors that are generally associated with peripartum challenges and HYK outcomes associated with shifts in nutrient partitioning reflective of negative energy balance. The diverse dataset described herein includes a cross section of farm size and production levels within which we could test our hypothesis. Cow data were contributed from herds of all sizes and assuredly represent different management practices and nutritional approaches. The dataset included a wide range of herd sizes with RHA analysis based on a minimum of 33, a maximum of 7512, and a median of 228 cows tested. This demonstrates that the very large farms were included but were not overrepresented in the dataset. Importantly, development and validation of the multiple linear regression models used to predict HYK within this dataset was within this same region [4]. Herds also varied in production with RHA milk production ranging 5500 to more than 18,000 kg of milk and RHA milk fat and protein percent ranging from 2.82 to 5.31 and 2.82 to 3.64%, respectively. The breadth of the dataset in cow and herd demographics provides a broader scope of interest for interpreting associations at the herd- and cow-level. Herd-level prevalence of pHYK was inversely associated with RHA protein percent, but not fat percent. Interestingly, pHYK decreased as RHA milk production increased within this dataset, suggesting that greater HYK prevalence is not a consequence of greater herd-level production.

Previous lactation performance and insults during the transition to lactation period can influence postpartum health and examination of herd demographics and events prior to HYK onset can provide insight into these potential risk factors. Within the current dataset, previous lactation SCC was greater for pHYK cows. Dry period length was greater for pHYK cows across lactation groups, which is consistent with past studies and has been suggested to be related to increased adiposity associated with longer dry periods [16,17]. Additionally, calving ease score was greater for cows with pHYK. Greater calving ease score and dystocia can create challenges at parturition leading to decreased feed intake and has been noted as a risk factor of clinical ketosis in some [18] but not all [19,20] analyses. There was also a greater calf mortality in calves born to pHYK first lactation cows. Stillbirths can contribute to maternal immune or energy challenges and, like dystocia, contributes to uterine disease, displaced abomasum, and culling risks [21]. Together, factors such as dry period length and calving challenges may increase HYK risk indirectly through increased body condition score prepartum or decreased feed intake peripartum. Although causality cannot be determined from this type of data, repeated associations between key factors such as days dry and dystocia support implementing management that would decrease these factors or more closely monitoring cows that fit these criteria.

One of the often-reported negative impacts of untreated HYK is decreased milk production [18,22]; however, this is not always observed when proactive diagnostic and treatment protocols are implemented [23,24]. Within the current data set, pHYK cows had lower milk yield which was not simply a product of herd milk average, since herds in the higher quartiles of RHA milk yield had a lower HYK prevalence. It has been previously suggested that the negative impact of HYK on milk yield is temporary, without having an overall impact on predicted 305 d milk yields [16]. Given that milk yield is reflective of so many factors (e.g., farm, genetics, diet, environment, detection and treatment approaches, etc.) it is challenging to determine the impact of HYK on short- or long-term milk yield from datasets that do not include all of these factors.

Given the association of HYK with energy balance, it is also important to consider milk components which greatly influence milk energy output. Specifically, milk fat represents half of the energy in milk [25] and the profile of FA within the milk can shed light on the source of milk fat [26,27]. Milk fat percent is commonly reported as being greater in cows with HYK [16,17,23,28], consistent with the data presented herein. When calculated, both fat-corrected and energy-corrected milk are greater in cows with HYK [23], which suggests that the impact of HYK on milk synthesis may reflect a shift in nutrient partitioning rather than generalized negative impacts on milk synthesis. Dynamic nutrient partitioning postpartum is a key component to optimizing metabolism and health [29,30]. Coordinated shifts to spare glucose may result in decreased milk yield while relying on the more abundant mobilized fat to maintain milk energy output.

Not only does milk fat yield increase, the profile of milk FA may shift postpartum when comparing cows with HYK or fatty liver [31,32]. When triglycerides are mobilized from adipose tissue postpartum, blood FA increase, and the blood FA profile resembles the FA profile of adipose tissue more closely. Circulating blood FA provide a precursor to milk fat synthesis so in addition to resulting in increased milk fat, increased adipose triglyceride mobilization can lead to a downstream shift in milk FA profile to reflect the change in milk fat precursors, as determined by gas chromatography [33,34,35]. High-throughput analysis of milk FA groups by FTIR analysis is being explored [27,36] and although not perfectly aligned with gas chromatography analysis, FTIR-based predicted FA can be grouped as de novo (<C16), mixed (C16), and preformed (>C16) and can provide insight into the physiology described above. Within the current dataset, the percent of milk de novo FA were lower, while the percent of mixed and preformed FA were greater in pHYK cows. This shift may be reflective of the increased fat mobilization that occurs postpartum and is associated with HYK. Interestingly, an early attempt to detect shifts in milk FTIR-determined FA groups corresponding to blood FA changes during HYK yielded the same numerical patterns, although not significant in that relatively small sample size [37]. Identification of clear patterns of FTIR-determined milk FA groups in the current analysis provides valuable insight into the use of these data relative to health and physiology on a larger high-throughput scale.

Within this data set, pHYK cows also had lower previous lactation ME protein percent and milk protein percent in the first milk test, which has been noted previously [16,28]. Milk protein percent was also decreased in cows diagnosed with HYK by blood diagnostics, blood BHB ≥ 1.2 mM, and immediately treated [23]. It is interesting that milk protein percent is decreased despite the presence, absence, or presumed mixture of HYK treatments. It was previously suggested that the inverse relationship between milk protein and HYK was primarily due to negative energy balance, given the positive association between energy balance and protein synthesis [17]; however, the association with a lower previous ME305 protein percent may suggest an additional connection. Alternatively, decreased ME305 milk protein may reflect patterns of lactational energy or protein imbalance that could influence metabolism or nutrient partitioning that favors gluconeogenesis from amino acids decreasing availability for milk protein synthesis, although neither have been directly examined. Unfortunately, milk components are not always reported within HYK experiments and therefore only a subset of HYK studies can be examined for these connections. Potential interactions between whole-body and tissue-level protein metabolism and metabolic state should be further explored in future work and milk composition should be analyzed and recorded in sample sets of this type.

In addition to milk energy components, milk SCC is a DHI-provided indicator of udder health and milk quality that is widely accepted and used by farms, management and clinical teams, and milk processors [38,39,40]. First test milk SCC and SCS were both greater for pHYK cows across lactations. Associations between elevated SCC and both body weight loss and ketosis have been identified previously and it has been proposed that the underlying greater negative energy balance may predispose cows to udder inflammation [41]. In addition to the association between ketosis and increased SCC, more direct markers of immune status, including activation of the inflammatory signaling pathways and increases in proinflammatory cytokines, have been observed during ketosis [42,43]. Quantification of dSCC has allowed for quantification of polymorphonuclear neutrophils and lymphocytes, relative to total SCC, and may provide further insight into intra-mammary infection [44,45]. Within the current dataset, dSCC was greater for pHYK in parity 2 and 4. Interestingly, the pattern of SCC and dSCC between pHYK and pNonHYK cows is not identical across parity in this data or in a previous study that examined two herds [45]. Direct research will be needed to determine if dSCC can provide additional value in understanding the type of local inflammation or infection or identifying chronic or persistent inflammatory cases.

Not all herds routinely record health events in a consistent, exportable manner; therefore, the current dataset was censored to include health events only from farms that recorded and provided health records. When combined with the nature of convenience sampling, these factors may artificially influence the prevalence of disorders presented. Additionally, since some cows contributed several lactations worth of data, maintaining cows with lower risk in the herd may have diluted lactational prevalence than reported in randomly sampled herds [46]. Relationships between comorbid transition cow health disorders including HYK, DA, and retained placenta within the current dataset are consistent or lower than previous reports [18,20,21,22,47], despite the aforementioned potential limitations to health records retrieval. In contrast, recorded events of ketosis were lower than expected based on incidence during research with well-defined detection programs [4,5,6] and the predictions implemented within this research, which highlights the low detection of sub-clinical disorders and reiterates the need for data-based detection and prediction tools. Another limitation to using farm-reported dairy management software is that all farms record health events differently creating limitations in analysis. In contrast to on-farm records, what is recorded in herd management software is often recorded as treatments rather than disorder, recorded using names or codes that are not compiled into larger datasets, and reflect non-sampling error. For example, across farms ketosis (subclinical and clinical) is entered as KET, SCK (sub-clinical ketosis), CK (clinical ketosis), PG (propylene glycol), DEX (dextrose), K (ketosis), BHB, etc. and some farms record as a protocol rather than an event, especially considering limitations on lifetime event entries in some programs. In all cases, specific treatment is not always evident from the recorded event and therefore cannot be accounted for in research based on farm-recorded data. This is further confounded for sub-clinical disorders because when dairy management software event data is exported, it is not linked to the presence, absence, or compliance of a detection program and by definition, sub-clinical disorders do not have readily observable symptoms. Together these reasons likely explain the low agreement of farm-reported cases of ketosis within pHYK cows and highlight the value of low-input, low-expense detection methods for sub-clinical disorders. The need for a unified and consistent recording system has been widely recognized [2,48,49,50], but until formats can be broadly applied and merged downstream are adopted, these will continue to be limitations.

As has been observed previously, there was increased incidence of farm-reported DA in pHYK cows, although occurrence of DA was later in lactation for pHYK cows than pNonHYK cows. Across cows with completed lactations, days open and number of artificial inseminations were both greater for pHYK cows. Reproductive inefficiencies not only represent an economic challenge, but also perpetuate the cycle of later breeding and longer dry periods: cows that are not bred back quickly often have longer lactations and dry periods, presenting HYK risk factors going into the next lactation. Despite the limitations noted above, the patterns for comorbidities and culling noted here are consistent with what has been observed previously [18,20,21,22,47]. Here, and elsewhere, caution should be used in interpreting associations between comorbidities as they do not indicate causality and analysis does not include a longitudinal aspect and timing of the postpartum events varies between prior to, concurrent with, or subsequent to the pHYK event across cows within the dataset. As noted above regarding HYK treatment, the treatment regimens for comorbidities are not included in the analysis and therefore can influence the associations. Despite these limitations, understanding disease etiology through more intense research studies [22,23,51,52] and aligning that knowledge with on-farm outcomes such as those presented here allows for a more robust understanding of the impact of metabolic disorders.

Ultimately, occurrence of multiple health events during the transition to lactation period is a contributor to early lactation culling, with decisions often reflecting both health events and production variables. Involuntary culling is often listed as an outcome of metabolic disorders and within the current dataset is at least twice as great within pHYK cows across lactations. In first lactation animals, culling was 2.4 times greater for pHYK cows. Involuntary culling can put constraints on voluntary culling and limit profitability, especially in cases of first-lactation cows that have not recovered heifer raising costs. Time to culling for pHYK cows was longer than pNonHYK cows which is consistent with timing for DA. Exploration of reasons provided for culling reveals death and injury/disease being cited more often for pHYK cows than pNonHYK cows.

A unique aspect of this dataset is the ability to analyze production for pHYK and pNonHYK cows within the culled and retained cohorts which can provide insight into farm-level decisions that may curtail negative outcomes. For example, cows sold for dairy are not viewed as negatively as injury/disease or death; however, they still represent a farm decision to not retain that cow in the herd which provides useful information. This may be evident by similar peak milk yield between pHYK and pNonHYK in retained, but not culled, subsets of cows which likely reflects the influence that a peak milk has on culling decisions. Despite this, ME milk yield portrayed a production disparity by HYK status regardless of culling decisions. Across first-test ME milk yield and protein, pHYK cows that were retained in the herd had significantly lower yields than pNonHYK cows, although the production gap is less obvious in second and greater lactation pHYK cows. This suggests that the culling decisions successfully sorted out cows with the greatest production disparity, even if pHYK was not the only contributor. Retained pHYK cows had greater ME fat yield, yet this was not observed in culled pHYK cows in first and second lactations. This is an interesting finding, especially within context of the relative value verses harm of mobilized fat during the transition period [29,30,53]. As discussed above, milk fat yield is a downstream reflector of mobilized fat but the contrasting pattern of retained verses culled cows suggests that the mobilized fat may not have been detrimental and could, as postulated above, be an aspect of coordinated energy partitioning. An alternative suggestion is that component yield may have a weighted value in culling decisions on farm.

Genetic selection is an important component of comprehensive herd management to improve cow health and productivity. Previous research has supported the role of genomic selection in both reducing HYK and genome-guided management practices [54]. Genome-wide association studies have identified associations that may provide value as markers of HYK risk [55,56,57]. Within the current analysis, the lower genetic resistance (or PTA) for ketosis observed for pHYK cows confirms that the predictive models and genetic evaluations are mutually identifying cows experiencing or more disposed to a case of ketosis. On a broader scope, associating trait PTA to pHYK status could provide additional evidence of factors that predispose cows to HYK before pHYK diagnosis; however, it is important to remember that these PTA are informed by phenotypes potentially realized after a disease incidence. Although not observed in a previous smaller dataset [23], milk and milk component yield PTA were lower for pHYK cows across parities, in agreement with the phenotypic observations described herein. This pattern of congruency is maintained for the lower PTA for daughter pregnancy rate, productive life, and SCS for pHYK, which was reflected in the phenotypic observations of fertility (DA and artificial insemination services), herd retention, and SCS. These associations either provide further evidence for the discussed relationships or suggest value in accounting for metabolic health in evaluation of the traits. Either way, these findings support previous suggestions [2] to use genetic markers or PTAs in identifying individual cows that may have increased risk for HYK or other metabolic disorders to improve management practices.

## 5. Conclusions

In addition to providing a diagnostic and monitoring value to farms, broad adoption of data-based predictions of health incidences generates large datasets that can be used for epidemiological examination of outcomes in real-world settings. Application of predictions for HYK in the Midwest region of the United States has allowed for population-level exploration of production and health outcomes although it must be noted that HYK, other disease incidence, and treatments cannot be verified in a dataset of this size. As hypothesized, and consistent with past research, factors that represent a challenging peripartum period such as previous lactation SCC, days dry, and dystocia, are greater for cows subsequently pHYK. Milk yield and milk protein percent were less at first milk test and milk fat percent, energy-corrected milk yield, and SCC were greater for pHYK at first milk test. Novel findings include that mixed and preformed FA were greater in pHYK across parities, and differential SCC is greater in pHYK in some parities. Culling within 60 d postpartum was greater for pHYK, and ME milk yield and protein yield were less for pHYK both within the culled and retained subsets of cows. Conversely, ME fat yield was greater for retained pHYK cows; however, this was only true in third or greater culled HYK cows. Although pHYK was associated with greater incidence of DA, it should be noted that information regarding treatments for any health events was not included in the analysis.

Overall, data are consistent with previous outcomes associated with HYK. The range of farm sizes and production levels increases the value of data inference. Although HYK is sometimes attributed to high levels of production, it was interesting to find that within this dataset, pHYK linearly decreased as farm RHA milk yield increased. More recent advances in milk FA groups and differential SCC provide additional insight and warrant future investigation on characterizing postpartum fat metabolism and inflammation. Differences between pHYK and pNonHYK cows in peak milk yield and ME milk fat yield, and less severe differences between ME milk yield and ME milk protein between culled and retained cohorts, supports that on-farm culling practices are curtailing pHYK outcomes. Future work should further explore the dichotomy of decreased milk yield and protein content but increased energy-corrected milk yield in pHYK cows as a way to understand the role of nutrient partitioning in etiology of HYK and other metabolic disorders.

## Figures and Tables

**Table 1:** Descriptive Statistics of Test Day Samples Collected from Cows between 5 and 20 Days in Milk and Classified into Predicted Hyperketonemic (pHYK) or Predicted Nonhyperketonemic (pNonHYK) Using Multiple Linear Regression ^1^.

Variable	N, Denominator ^2^	Mean ^3^	SD ^4^	Min ^5^	Q1 ^6^	Median	Q3 ^7^	Max ^8^
All Records	240,714	—	—	—	—	—	—	—
Records/Cow ^9^	174,690	1.4	0.7	1.0	1.0	1.0	2.0	6.0
Records/Herd ^10^	335	718.6	1328.0	11.0	116.0	255.0	641.0	13,204.0
Cows/Herd ^11^	335	521.5	949.1	11.0	91.5	179.0	468.5	9550.0
Primiparous Records	88,782	—	—	—	—	—	—	—
Records/Cow	88,782	1.0	0.0	1.0	1.0	1.0	1.0	1.0
Records/Herd	333	266.6	499.5	1.0	42.0	97.0	232.0	5091.0
Cows/Herd	333	266.6	499.5	1.0	42.0	97.0	232.0	5091.0
Records %, Herd ^12^	—	36.9	13.8	0.0	31.8	36.5	40.6	99.5
Multiparous Records	151,932	—	—	—	—	—	—	—
Records/Cow	115,745	1.3	0.6	1.0	1.0	1.0	2.0	6.0
Records/Herd	335	453.5	835.4	5.0	76.5	163.0	403.0	8113.0
Cows/Herd	335	345.5	632.6	5.0	59.5	122.0	311.5	6318.0
Records %, Herd	—	63.1	13.8	0.5	59.4	63.5	68.3	100.0
pNonHYK Records	202,659	—	—	—	—	—	—	—
Records/Cow	155,646	1.3	0.6	1.0	1.0	1.0	1.0	6.0
Records/Herd	335	605.0	1127.5	9.0	95.0	211.0	545.0	10,935.0
Cows/Herd	335	464.6	853.6	9.0	80.5	160.0	426.0	8516.0
Records %, Herd	—	83.8	7.6	52.1	79.5	85.3	89.2	97.7
pHYK Records	38,055	—	—	—	—	—	—	—
Records/Cow	34,427	1.1	0.3	1.0	1.0	1.0	1.0	4.0
Records/Herd	335	113.6	216.7	1.0	16.5	40.0	105.5	2269.0
Cows/Herd	335	102.8	194.5	1.0	16.0	36.0	94.0	2063.0
Records %, Herd	—	16.2	7.6	2.3	10.8	14.7	20.5	47.9

^1^ Predictions for hyperketonemia were determined by applying previously published multiple linear regression models [4] to predict a continuous blood ß-hydroxybutyrate and categorized as pHYK when predicted concentrations were greater than or equal to 1.2 mM.; ^2^ Number of records in each subset of the data; ^3^ Arithmetic mean; ^4^ Standard deviation; ^5^ Minimum; ^6^ First quartile (bottom 25%) threshold; ^7^ Third quartile (top 25%) threshold; ^8^ Maximum; ^9^ Number of records from unique cows; ^10^ Number of records from unique herds; ^11^ Number of cows from unique herds; ^12^ Percent of record type of the herd.

**Table 2:** Overall and Rolling Herd Average (RHA) Quartile Descriptive Statistics of Herds Providing Test Day Samples.

Variable	Mean ^1^	SD ^2^	Min ^3^	Q1 ^4^	Median	Q3 ^5^	Max ^6^
All Records (11,539 Records; 321 Herds)	—	—	—	—	—	—	—
Records per Herd	36.0	24.1	1.0	15.0	30.0	62.0	72.0
Cows Tested, RHA	467.7	681.5	32.8	125.3	228.0	562.8	7511.7
Postpartum Cows Tested ^7^	220.0	327.9	11.0	57.0	105.0	250.0	3878.0
Predicted HYK, % ^8^	15.9	8.4	0.0	9.8	14.9	20.7	58.8
RHA Milk, kg	12,125.8	1729.3	5539.3	11,136.6	12,265.6	13,264.6	18,196.3
RHA Milk Fat, kg	465.1	68.3	225.0	421.4	466.3	513.0	684.9
RHA Milk Fat, %	3.8	0.2	2.8	3.7	3.8	4.0	5.3
RHA Milk Protein, kg	376.2	51.9	176.0	345.6	380.6	409.6	536.2
RHA Milk Protein, %	3.1	0.1	2.8	3.0	3.1	3.2	3.6
RHA Quartile 1 (2885 Records; 158 Herds)	—	—	—	—	—	—	—
Records per Herd	18.3	17.2	1.0	5.0	13.0	24.8	69.0
Cows Tested, RHA	197.0	507.6	34.0	82.5	118.2	174.4	5596.1
Postpartum Cows Tested	84.3	195.5	11.0	39.0	56.0	80.0	2914.0
Predicted HYK, %	16.6	9.4	0.0	9.8	15.4	22.2	58.8
RHA Milk, kg	9870.7	1119.1	5539.3	9467.8	10,151.8	10,665.3	11,136.1
RHA Milk Fat, kg	382.6	45.5	225.0	362.4	388.7	412.8	585.1
RHA Milk Fat, %	3.9	0.2	3.1	3.7	3.9	4.0	5.3
RHA Milk Protein, kg	309.5	35.0	176.0	297.1	318.0	332.9	372.0
RHA Milk Protein, %	3.1	0.1	2.9	3.1	3.1	3.2	3.6
RHA Quartile 2 (2884 Records; 147 Herds)	—	—	—	—	—	—	—
Records per Herd	19.6	16.5	1.0	6.0	15.0	28.0	66.0
Cows Tested, RHA	378.8	730.9	32.8	113.2	180.7	296.0	7511.7
Postpartum Cows Tested	178.4	350.7	11.0	54.8	83.0	140.0	3878.0
Predicted HYK, %	15.8	8.4	0.0	9.7	14.9	20.6	58.3
RHA Milk, kg	11,746.0	323.8	11,137.0	11,482.6	11,745.3	12,039.4	12,264.7
RHA Milk Fat, kg	447.5	28.1	339.7	429.1	445.9	462.7	590.1
RHA Milk Fat, %	3.8	0.2	2.8	3.7	3.8	3.9	5.3
RHA Milk Protein, kg	365.4	14.0	327.0	355.6	364.7	375.1	414.1
RHA Milk Protein, %	3.1	0.1	2.8	3.1	3.1	3.2	3.4
RHA Quartile 3 (2885 Records; 142 Herds)	—	—	—	—	—	—	—
Records per Herd	20.0	16.1	1.0	6.8	17.0	29.3	70.0
Cows Tested, RHA	630.8	771.1	35.7	178.7	387.0	727.8	5227.7
Postpartum Cows Tested	297.1	381.2	11.0	80.0	159.0	323.0	2524.0
Predicted HYK, %	16.4	7.6	0.0	11.1	15.6	20.7	46.5
RHA Milk, kg	12,745.0	290.3	12,265.6	12,495.6	12,726.4	12,994.5	13,264.4
RHA Milk Fat, kg	489.3	31.0	361.1	468.1	488.1	508.0	622.0
RHA Milk Fat, %	3.8	0.2	2.9	3.7	3.8	4.0	4.8
RHA Milk Protein, kg	394.6	14.6	347.5	385.1	394.2	403.7	439.1
RHA Milk Protein, %	3.1	0.1	2.8	3.0	3.1	3.2	3.4
RHA Quartile 4 (2885 Records; 100 Herds)	—	—	—	—	—	—	—
Records per Herd	28.9	21.3	1.0	10.8	23.0	42.3	72.0
Cows Tested, RHA	664.3	570.5	29.4	324.4	527.6	756.3	3418.8
Postpartum Cows Tested	320.1	296.1	11.0	122.0	251.0	384.0	2156.0
Predicted HYK, %	14.9	7.8	0.0	8.7	13.5	19.3	48.3
RHA Milk, kg	14,141.4	848.1	13,264.8	13,550.6	13,908.5	14,453.3	18,196.3
RHA Milk Fat, kg	540.9	37.5	430.9	515.3	537.1	563.4	684.9
RHA Milk Fat, %	3.8	0.3	3.1	3.7	3.8	4.0	4.9
RHA Milk Protein, kg	435.3	27.1	382.4	415.0	429.6	450.0	536.2
RHA Milk Protein, %	3.1	0.1	2.9	3.0	3.1	3.2	3.4

^1^ Arithmetic mean; ^2^ Standard deviation; ^3^ Minimum; ^4^ First quartile (bottom 25%) threshold; ^5^ Third quartile (top 25%) threshold; ^6^ Maximum; ^7^ Samples collected between 5 and 20 days in milk; ^8^ Predictions for hyperketonemia were determined by applying previously published multiple linear regression models [4] to predict a continuous blood ß-hydroxybutyrate and categorized as pHYK when predicted concentrations were greater than or equal to 1.2 mM.

**Table 3:** Parity by Predicted Hyperketonemia (HYK) Status Interaction on Management Outcomes and Genetic Predictions for Cows between 5 and 20 Days in Milk and Classified into Predicted Hyperketonemic (pHYK) or Predicted Nonhyperketonemic (pNonHYK) Using Multiple LINEAR regression ^1^.

Lactation ^2^												
Variable	1		2		3		4		5+			*p*-Value
	pNonHYK	pHYK	pNonHYK	pHYK	pNonHYK	pHYK	pNonHYK	pHYK	pNonHYK	pHYK	SEM	Health × Parity ^3^
Cull within 60d, %	5.7	13.7 ***	3.4	7.6 ***	4.1	10.5 ***	5.6	11.8 ***	7.4	13.5 ***	0.95	0.003
Days open, d	120.9	133.0 ***	126.9	132.6 ***	128.7	132.0 ***	128.6	132.8 ***	132.4	141.2 ***	2.33	<0.001
AI ^4^ to Conception	2.0	2.1 ***	2.1	2.2 ***	2.2	2.2 **	2.1	2.3 ***	2.2	2.4 ***	0.04	<0.001
PTA ^5^ milk, kg	−14.1	−12.0	−58.5	−66.4	−93.1	−106.6 *	−132.2	−143.9 *	−186.3	−207.7 ***	10.0	0.02
PTA DPR ^6^	−0.27	−0.47 ***	−0.19	−0.33 ***	−0.06	−0.17 ***	0.12	0.05 *	0.33	0.36	0.05	<0.001
PTA productive life	0.12	−0.34	−0.12	−0.39	−0.20	−0.47 *	−0.23	−0.43 *	−0.22	−0.28 ***	0.07	<0.001
PTA SCS, score	2.99	3.01 ***	3.00	3.01 ***	3.01	3.02 ***	3.02	3.03 ***	3.02	3.03 ***	0.004	<0.001
PTA net merit	5.48	−22.95 ***	−45.39	−63.04 ***	−82.78	−103.37 ***	−119.86	−136.83 ***	−165.74	−176.40 *	7.18	0.01
PTA ketosis	0.05	−0.11 ***	−0.09	−0.18 ***	−0.19	−0.29 ***	−0.30	−0.38 ***	−0.41	−0.46 ***	0.02	<0.001

^1^ Blood ß-hydroxybutyrate (BHB) was predicted based on test day milk sample and production variables collected between 5 to 20 days in milk. Predictions were classified as predicted HYK (pHYK; predicted BHB ≥ 1.2 mM) or not (pNonHYK; predicted BHB < 1.2 mM) for analysis of records; ^2^ Records per lactation 1: 88,782; 2: 67,327; 3: 43,790; 4: 23999; 5+: 16,816; ^3^ Interaction of predicted health and parity; ^4^ Artificial inseminations; ^5^ Predicted transmitting ability; provided by Center for Dairy Cattle Breeding; ^6^ Daughter pregnancy rate; Superscript symbols indicate simple effect of classification (***, *p* < 0.001; **, 0.001 < p < 0.01; *, 0.01 < *p* ≤ 0.05) within parity.

## Data Availability

Restrictions apply to the availability of these data. Data was obtained from VAS (Madison, WI, USA) and are available from the authors with the permission of VAS.

## References

[1] Cabrera V.E., Barrientos-Blanco J.A., Delgado H., Fadul-Pacheco L. (2020). Symposium Review: Real-Time Continuous Decision Making Using Big Data on Dairy Farms. J. Dairy Sci..

[2] Pralle R.S., White H.M. (2020). Symposium Review: Big Data, Big Predictions: Utilizing Milk Fourier-Transform Infrared and Genomics to Improve Hyperketonemia Management. J. Dairy Sci..

[3] Halachmi I., Guarino M., Bewley J., Pastell M. (2018). Smart Animal Agriculture: Application of Real-Time Sensors to Improve Animal Well-Being and Production. Annu. Rev. Anim. Biosci..

[4] Chandler T.L., Pralle R.S., Dórea J.R.R., Poock S.E., Oetzel G.R., Fourdraine R.H., White H.M. (2018). Predicting Hyperketonemia by Logistic and Linear Regression Using Test-Day Milk and Performance Variables in Early-Lactation Holstein and Jersey Cows. J. Dairy Sci..

[5] Suthar V.S., Canelas-Raposo J., Deniz A., Heuwieser W. (2013). Prevalence of Subclinical Ketosis and Relationships with Postpartum Diseases in European Dairy Cows. J. Dairy Sci..

[6] Santschi D.E., Lacroix R., Durocher J., Duplessis M., Moore R.K., Lefebvre D.M. (2016). Prevalence of Elevated Milk β-Hydroxybutyrate Concentrations in Holstein Cows Measured by Fourier-Transform Infrared Analysis in Dairy Herd Improvement Milk Samples and Association with Milk Yield and Components. J. Dairy Sci..

[7] McArt J.A.A., Nydam D.V., Overton M.W. (2015). Hyperketonemia in Early Lactation Dairy Cattle: A Deterministic Estimate of Component and Total Cost per Case. J. Dairy Sci..

[8] Pralle R.S., Weigel K.W., White H.M. (2018). Predicting Blood β-Hydroxybutyrate Using Milk Fourier Transform Infrared Spectrum, Milk Composition, and Producer-Reported Variables with Multiple Linear Regression, Partial Least Squares Regression, and Artificial Neural Network. J. Dairy Sci..

[9] Rutten M.J.M., Bovenhuis H., Hettinga K.A., van Valenberg H.J.F., van Arendonk J.A.M. (2009). Predicting Bovine Milk Fat Composition Using Infrared Spectroscopy Based on Milk Samples Collected in Winter and Summer. J. Dairy Sci..

[10] Rutten M.J.M., Bovenhuis H., Heck J.M.L., van Arendonk J.A.M. (2011). Predicting Bovine Milk Protein Composition Based on Fourier Transform Infrared Spectra. J. Dairy Sci..

[11] Van der Drift S.G.A., Jorritsma R., Schonewille J.T., Knijn H.M., Stegeman J.A. (2012). Routine Detection of Hyperketonemia in Dairy Cows Using Fourier Transform Infrared Spectroscopy Analysis of β-Hydroxybutyrate and Acetone in Milk in Combination with Test-Day Information. J. Dairy Sci..

[12] Denis-Robichaud J., Dubuc J., Lefebvre D., DesCôteaux L. (2014). Accuracy of Milk Ketone Bodies from Flow-Injection Analysis for the Diagnosis of Hyperketonemia in Dairy Cows. J. Dairy Sci..

[13] De Roos A.P.W., van den Bijgaart H.J.C.M., Hørlyk J., de Jong G. (2007). Screening for Subclinical Ketosis in Dairy Cattle by Fourier Transform Infrared Spectrometry. J. Dairy Sci..

[14] Chandler T.L., Pralle R.S., Oetzel G.R., Fourdraine R.H., White H.M. (2015). Development of a Ketosis Prevalence Detection Tool in Holstein Dairy Cows Based on Milk Component Data and Cow Test-Day Information. J. Dairy Sci..

[15] Borchers M.R., Bewley J.M. (2015). An Assessment of Producer Precision Dairy Farming Technology Use, Prepurchase Considerations, and Usefulness. J. Dairy Sci..

[16] Vanholder T., Papen J., Bemers R., Vertenten G., Berge A.C.B. (2015). Risk Factors for Subclinical and Clinical Ketosis and Association with Production Parameters in Dairy Cows in the Netherlands. J. Dairy Sci..

[17] Duffield T. (2000). Subclinical Ketosis in Lactating Dairy Cattle. Vet. Clin. N. Am. Food Anim. Pract..

[18] Duffield T.F., Lissemore K.D., McBride B.W., Leslie K.E. (2009). Impact of Hyperketonemia in Early Lactation Dairy Cows on Health and Production. J. Dairy Sci..

[19] Curtis C.R., Erb H.N., Sniffen C.J., Smith R.D., Kronfeld D.S. (1985). Path Analysis of Dry Period Nutrition, Postpartum Metabolic and Reproductive Disorders, and Mastitis in Holstein Cows1. J. Dairy Sci..

[20] Gröhn Y.T., Erb H.N., McCulloch C.E., Saloniemi H.S. (1989). Epidemiology of Metabolic Disorders in Dairy Cattle: Association Among Host Characteristics, Disease, and Production. J. Dairy Sci..

[21] Vergara C.F., Döpfer D., Cook N.B., Nordlund K.V., McArt J.A.A., Nydam D.V., Oetzel G.R. (2014). Risk Factors for Postpartum Problems in Dairy Cows: Explanatory and Predictive Modeling. J. Dairy Sci..

[22] McArt J.A.A., Nydam D.V., Oetzel G.R. (2012). Epidemiology of Subclinical Ketosis in Early Lactation Dairy Cattle. J. Dairy Sci..

[23] Rathbun F.M., Pralle R.S., Bertics S.J., Armentano L.E., Cho K., Do C., Weigel K.A., White H.M. (2017). Relationships between Body Condition Score Change, Prior Mid-Lactation Phenotypic Residual Feed Intake, and Hyperketonemia Onset in Transition Dairy Cows. J. Dairy Sci..

[24] McArt J.A.A., Nydam D.V., Oetzel G.R. (2012). A Field Trial on the Effect of Propylene Glycol on Displaced Abomasum, Removal from Herd, and Reproduction in Fresh Cows Diagnosed with Subclinical Ketosis. J. Dairy Sci..

[25] Bauman D.E., Currie W.B. (1980). Partitioning of Nutrients During Pregnancy and Lactation: A Review of Mechanisms Involving Homeostasis and Homeorhesis. J. Dairy Sci..

[26] Samková E., Špička J., Hanuš O., Roubal P., Pecová L., Hasoňová L., Smetana P., Klimešová M., Čítek J. (2020). Comparison of Fatty Acid Proportions Determined by Mid-Infrared Spectroscopy and Gas Chromatography in Bulk and Individual Milk Samples. Animals.

[27] Woolpert M.E., Dann H.M., Cotanch K.W., Melilli C., Chase L.E., Grant R.J., Barbano D.M. (2017). Management Practices, Physically Effective Fiber, and Ether Extract Are Related to Bulk Tank Milk de Novo Fatty Acid Concentration on Holstein Dairy Farms. J. Dairy Sci..

[28] Miettinen P.V.A. (1994). Relationship between Milk Acetone and Milk Yield in Individual Cows. J. Vet. Med. Ser..

[29] White H.M. (2020). ADSA Foundation Scholar Award: Influencing Hepatic Metabolism: Can Nutrient Partitioning Be Modulated to Optimize Metabolic Health in the Transition Dairy Cow?. J. Dairy Sci..

[30] Drackley J.K. (1999). ADSA Foundation Scholar Award. Biology of Dairy Cows during the Transition Period: The Final Frontier?. J. Dairy Sci..

[31] Rukkwamsuk T., Geelen M.J., Kruip T.A., Wensing T. (2000). Interrelation of Fatty Acid Composition in Adipose Tissue, Serum, and Liver of Dairy Cows during the Development of Fatty Liver Postpartum. J. Dairy Sci..

[32] Weld K.A., Oliveira R.C., Bertics S.J., Erb S.J., White H.M. (2020). Hepatic Pyruvate Carboxylase Expression Differed Prior to Hyperketonemia Onset in Transition Dairy Cows. PLoS ONE.

[33] Tessari R., Berlanda M., Morgante M., Badon T., Gianesella M., Mazzotta E., Contiero B., Fiore E. (2020). Changes of Plasma Fatty Acids in Four Lipid Classes to Understand Energy Metabolism at Different Levels of Non-Esterified Fatty Acid (NEFA) in Dairy Cows. Animals.

[34] Mann S., Nydam D.V., Lock A.L., Overton T.R., McArt J.A.A. (2016). Short Communication: Association of Milk Fatty Acids with Early Lactation Hyperketonemia and Elevated Concentration of Nonesterified Fatty Acids. J. Dairy Sci..

[35] Jorjong S., van Knegsel A.T.M., Verwaeren J., Bruckmaier R.M., Baets B.D., Kemp B., Fievez V. (2015). Milk Fatty Acids as Possible Biomarkers to Diagnose Hyperketonemia in Early Lactation. J. Dairy Sci..

[36] Barbano D.M., Melilli C., Overton T.R. Advanced Use of FTIR Spectra of Milk for Feeding and Health Management. Proceedings of the Cornell Nutrition Conference.

[37] Weld A.K., Oliveira R.C., Sailer K.J., Holdorf H.T., Bertics S.J., White H.M. (2018). Hyperketonemia Does Not Affect Proportional Uptake of Fatty Acids by the Mammary Gland. J. Dairy Sci..

[38] Schukken Y.H., Wilson D.J., Welcome F., Garrison-Tikofsky L., Gonzalez R.N. (2003). Monitoring Udder Health and Milk Quality Using Somatic Cell Counts. Vet. Res..

[39] Harmon R.J. (1994). Physiology of Mastitis and Factors Affecting Somatic Cell Counts. J. Dairy Sci..

[40] Schwarz D., Kleinhans S., Reimann G., Stückler P., Reith F., Ilves K., Pedastsaar K., Yan L., Zhang Z., Valdivieso M. (2020). Investigation of Dairy Cow Performance in Different Udder Health Groups Defined Based on a Combination of Somatic Cell Count and Differential Somatic Cell Count. Prev. Vet. Med..

[41] Van Straten M., Friger M., Shpigel N.Y. (2009). Events of Elevated Somatic Cell Counts in High-Producing Dairy Cows Are Associated with Daily Body Weight Loss in Early Lactation. J. Dairy Sci..

[42] Shen T., Li X., Loor J.J., Zhu Y., Du X., Wang X., Xing D., Shi Z., Fang Z., Li X. (2019). Hepatic Nuclear Factor Kappa B Signaling Pathway and NLR Family Pyrin Domain Containing 3 Inflammasome Is Over-Activated in Ketotic Dairy Cows. J. Dairy Sci..

[43] Pralle R.S., Li W., White H.M. (2020). Hepatic Differential Gene Expression of Cows Clustered by Postpartum Metabolites: A Model for Susceptibility to Lipid-Related Metabolic Disorders. J. Dairy Sci..

[44] Damm M., Holm C., Blaabjerg M., Bro M.N., Schwarz D. (2017). Differential Somatic Cell Count—A Novel Method for Routine Mastitis Screening in the Frame of Dairy Herd Improvement Testing Programs. J. Dairy Sci..

[45] Kirkeby C., Toft N., Schwarz D., Farre M., Nielsen S.S., Zervens L., Hechinger S., Halasa T. (2020). Differential Somatic Cell Count as an Additional Indicator for Intramammary Infections in Dairy Cows. J. Dairy Sci..

[46] Economic Research Service Milk Cost of Production Estimates. http://www.ers.usda.gov/data-products/milk-cost-of-production-estimates/.

[47] LeBlanc S.J., Leslie K.E., Duffield T.F. (2005). Metabolic Predictors of Displaced Abomasum in Dairy Cattle. J. Dairy Sci..

[48] McConnel C.S., McNeil A.A., Hadrich J.C., Lombard J.E., Heller J., Garry F.B. (2018). A Comparison of a Novel Time-Based Summary Measure of Dairy Cow Health against Cumulative Disease Frequency. Irish Vet. J..

[49] Gaddis K.L.P., Cole J.B., Clay J.S., Maltecca C. (2014). Genomic Selection for Producer-Recorded Health Event Data in US Dairy Cattle. J. Dairy Sci..

[50] Mörk M., Lindberg A., Alenius S., Vågsholm I., Egenvall A. (2009). Comparison between Dairy Cow Disease Incidence in Data Registered by Farmers and in Data from a Disease-Recording System Based on Veterinary Reporting. Prev. Vet. Med..

[51] McArt J.A.A., Nydam D.V., Oetzel G.R., Overton T.R., Ospina P.A. (2013). Elevated Non-Esterified Fatty Acids and β-Hydroxybutyrate and Their Association with Transition Dairy Cow Performance. Vet. J..

[52] Pralle R.S., Erb S.J., Holdorf H.T., White H.M. (2021). Greater Liver PNPLA3 Protein Abundance in Vivo and in Vitro Supports Lower Triglyceride Accumulation in Dairy Cows. Sci. Rep..

[53] Grummer R.R. (2008). Nutritional and Management Strategies for the Prevention of Fatty Liver in Dairy Cattle. Vet. J..

[54] Weigel K.A., Pralle R.S., Adams H., Cho K., Do C., White H.M. (2017). Prediction of Whole-genome Risk for Selection and Management of Hyperketonemia in Holstein Dairy Cattle. J. Anim. Breed. Genet..

[55] Pralle R.S., Schultz N.E., White H.M., Weigel K.A. (2020). Hyperketonemia GWAS and Parity-Dependent SNP Associations in Holstein Dairy Cows Intensively Sampled for Blood β-Hydroxybutyrate Concentration. Physiol. Genom..

[56] Nayeri S., Schenkel F., Fleming A., Kroezen V., Sargolzaei M., Baes C., Cánovas A., Squires J., Miglior F. (2019). Genome-Wide Association Analysis for β-Hydroxybutyrate Concentration in Milk in Holstein Dairy Cattle. BMC Genet..

[57] Yan Z., Huang H., Freebern E., Santos D.J.A., Dai D., Si J., Ma C., Cao J., Guo G., Liu G.E. (2020). Integrating RNA-Seq with GWAS Reveals Novel Insights into the Molecular Mechanism Underpinning Ketosis in Cattle. BMC Genom..

